# Genetic Relationship in *Cicer* Sp. Expose Evidence for Geneflow between the Cultigen and Its Wild Progenitor

**DOI:** 10.1371/journal.pone.0139789

**Published:** 2015-10-08

**Authors:** Ruth van Oss, Shahal Abbo, Ravit Eshed, Amir Sherman, Clarice J. Coyne, George J. Vandemark, Hong-Bin Zhang, Zvi Peleg

**Affiliations:** 1 The Robert H. Smith Institute of Plant Sciences and Genetics in Agriculture, The Levi Eshkol School of Agriculture, The Hebrew University of Jerusalem, Rehovot, Israel; 2 Genomic unit Plant Sciences Institute, Agricultural Research Organization (ARO)-Volcani Center, Bet Dagan, Israel; 3 USDA-ARS Western Regional Plant Introduction Station, Mail Stop 646402, Washington State University, Pullman, Washington, United States of America; 4 Department of Soil and Crop Sciences and Institute for Plant Genomics and Biotechnology, Texas A & M University, College Station, Texas, United States of America; National Institute of Plant Genome Research (NIPGR), INDIA

## Abstract

There is a debate concerning mono- or poly-phyletic origins of the Near Eastern crops. In parallel, some authors claim that domestication was not possible within the natural range of the wild progenitors due to wild alleles flow into the nascent crops. Here we address both, the mono- or poly-phyletic origins and the domestications within or without the natural range of the progenitor, debates in order to understand the relationship between domesticated chickpea (*Cicer arietinum* L.) and its wild progenitor (*C*. *reticulatum* Ladizinsky) with special emphasis on its domestication centre in southeastern Turkey. A set of 103 chickpea cultivars and landraces from the major growing regions alongside wild accessions (*C*. *reticulatum*, *C*. *echinospermum* P.H Davis and *C*. *bijugum* K.H. Rech) sampled across the natural distribution range in eastern Turkey were genotyped with 194 SNPs markers. The genetic affinities between and within the studied taxa were assessed. The analysis suggests a mono-phyletic origin of the cultigen, with several wild accession as likely members of the wild stock of the cultigen. Clear separation between the wild and domesticated germplasm was apparent, with negligible level of admixture. A single *C*. *reticulatum* accession shows morphological and allelic signatures of admixture, a likely result of introgression. No evidence of geneflow from the wild into domesticated germplasm was found. The traditional farming systems of southeaster Turkey are characterized by occurrence of sympatric wild progenitor—domesticated forms of chickpea (and likewise cereals and other grain legumes). Therefore, both the authentic crop landraces and the wild populations native to the area are a unique genetic resource. Our results grant support to the notion of domestication within the natural distribution range of the wild progenitor, suggesting that the Neolithic domesticators were fully capable of selecting the desired phenotypes even when facing rare wild-domesticated introgression events.

## Introduction

A major topic in plant domestication studies concerns the issue of mono *versus* polyphyletic origin of crop plants (e.g., [1]). While for certain crops like maize [2], potato [3] and sunflower [4–6] there is a consensus around monophyletic origins, in other crops like rice this question is being debated due to conflicting results and interpretations (e.g., [7–11]). This issue is highly important because discussions concerning mono- or poly-phyletic origin of crop plants can hardly be disentangled from other aspects of the multidisciplinary study of plant domestication. These include (but not limited to) the time frame, the geographic location and human initiative underlying plant domestication (e.g., [12–23]).

While early studies aimed at identification of the wild stocks of crop plants were based mainly on classical botanic, genetic and cytogenetic tools (e.g., [1, 24–26]), the advent of genome wide DNA markers screening have opened new possibilities to identify the wild stocks of crop plants. The seminal work of Heun et al. [27] suggesting a monophyletic and localised domestication of einkorn wheat provoked a long (over 15 years) debate concerning the mode of origin of the Near Eastern founder crops (e.g., [14, 22, 23, 28–37]). While for certain crops like rice and wheat the debate relies on large databases, for other crops comprehensive information is still lacking e.g., Allaby et al. [38] for flax, and Chapman et al. [39] for safflower, or practically non-existent for chickpea.

Most wild progenitors of the Near Eastern grain crops have quite extensive natural distribution [40, 41]. In theory, the potential for geographically diffused domestication pattern as suggested by Weiss et al. [42] or Willcox [43] is greater in crops with wild progenitors spanning a relatively wide distribution like wheat, barley, pea or lentil. However, in the case of the wild progenitor of chickpea (*Cicer reticulatum* Ladizinsky) with its very limited natural range [44] the potential for multiple domestication events is much smaller. Indeed, even the advocates of the multiple (polyphyletic) domestication scenario in the Near East, accept that chickpea may have been domesticated only once [33]. Still, if reconstructions based on independent embarkation on plant cultivation-domestication truly represent the occurrences in the Neolithic Near East, one would expect to detect a polyphyletic signal by screening a wide range of wild and domesticated chickpea germplasm.

In the current study, we have used an SNP based genotyping platform [45] to screen a collection of wild *Cicer* accessions from diverse habitats across its natural range in southeastern Turkey alongside a collection of domesticated chickpea cultivars from its major growing regions in an attempt to trace the wild stock of domesticated chickpea. While the genetic relations between domesticated chickpea and the wild species may suggest a monophyletic origin, the overall relatedness pattern may raise questions concerning the nature of genetic data required for reliable detection of the ancestral wild stocks of crop plants.

## Materials and Methods

### Plant material and DNA isolation

**Fig 1:**
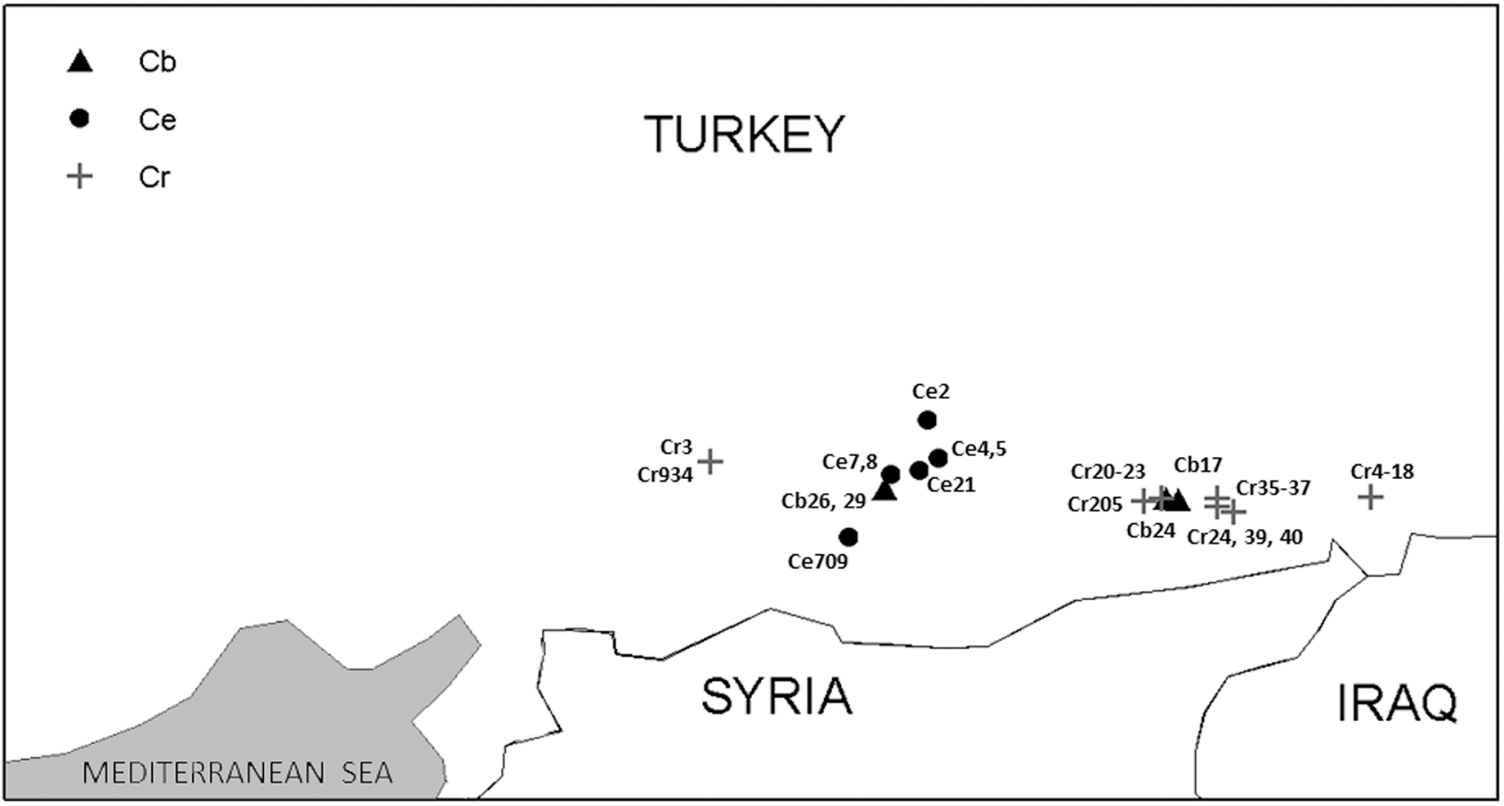
Geographical locations of the sampled wild populations employed in this study.

A diverse germplasm set of 103 *Cicer* genotypes, including 57 chickpea cultivars, 32 accessions of *C*. *reticulatum*, 7 accessions of *C*. *echinospermum*, 6 accessions of *C*. *bijugum* and one accession of *C*. *cuneatum* Hochst. ex Rich. (Fig 1; S1 Table) was used for this study. Due to its unique ecology, morphology and its remote phylogenetic relations with domesticated chickpea [46, 47] we chose *C*. *cuneatum* as an out-group. Seeds of chickpea cultivars were obtained from ICRISAT [48] and the USDA grain legume germplasm repository, Pullman WA, USA. The cultivar collection represents most major growing countries in the world. Additional domesticated lines were chosen from our working collection. Wild *Cicer* accessions were obtained from Dr. F.J. Muehlbauer (USDA, Pullman WA, USA), from Prof. G. Ladizinsky (Hebrew University, Rehovot, Israel) and our working collection.

Fresh leaf tissues (~200 mg) from two months old greenhouse-grown plants were used for DNA extraction by CTAB method, Follow RNase treatment. A NanoDrop^®^ ND1000 Spectrophotometer (NanoDrop Technologies, Inc., Wilmington, DE, USA) was used to measure the DNA concentration.

### Genotyping

The SNP markers were selected from the Chickpea KASPar (Competitive Allele Specific PCR) Assay Markers (CKAMs) developed by Hiremath et al. [45] KBioscience (http://www.kbioscience.co.uk). From the 2,005 CKAMs, a sub-set of 194 SNPs was identified following a preliminary polymorphism screen to cover all eight linkage groups. The genotyping was conducted at LGC Genomics.

### Genetic analysis

Individual pairwise genetic distances [49] were calculated for all markers. A principal coordinate analysis (PCoA) was performed on the markers data set with GENEALEX 6.5 (Genetic Analysis in Excel) software[49]. Analysis of molecular variance (AMOVA) was employed to estimate the variance between species and among accessions within species with 1000 bootstrap replicates.

The consensus unrooted tree of all the 103 *Cicer* genotypes was calculated in order to illustrate the level of relatedness between genotypes. The resulting distance matrix was subjected to sequential agglomerative hierarchical nested (SAHN) clustering using unweighted pair-group method analysis (UPGMA) as implemented in the PowerMarker software [50]. Bootstrapping over loci with 1000 replications was carried out to assess the strength of the evidence for the branching patterns in the resulting UPGMA tree. The consensus UPGMA tree with bootstrap values was reconstructed by the consensus program of PHYLIP (the PHYLogeny Inference Package) and displayed using the FigTree Ver. 1.4.2 software (http://tree.bio.ed.ac.uk/software/figtree).

The STRUCTURE 2.3.4 program [51] was used to analyse and cluster the studied genotypes. This program implements a model-based clustering method assigning individuals to clusters and identifying migrants and individuals resulting from admixture [51]. The number of clusters (*K*) was set from 1 to 8. Each *K* was replicated 10 times for 10,000 iterations after a burn-in period of 100,000. An admixture model was employed in which the fraction of ancestry from each cluster is estimated for each species.

## Results

### The relationship between the domesticated and wild species

**Fig 2:**
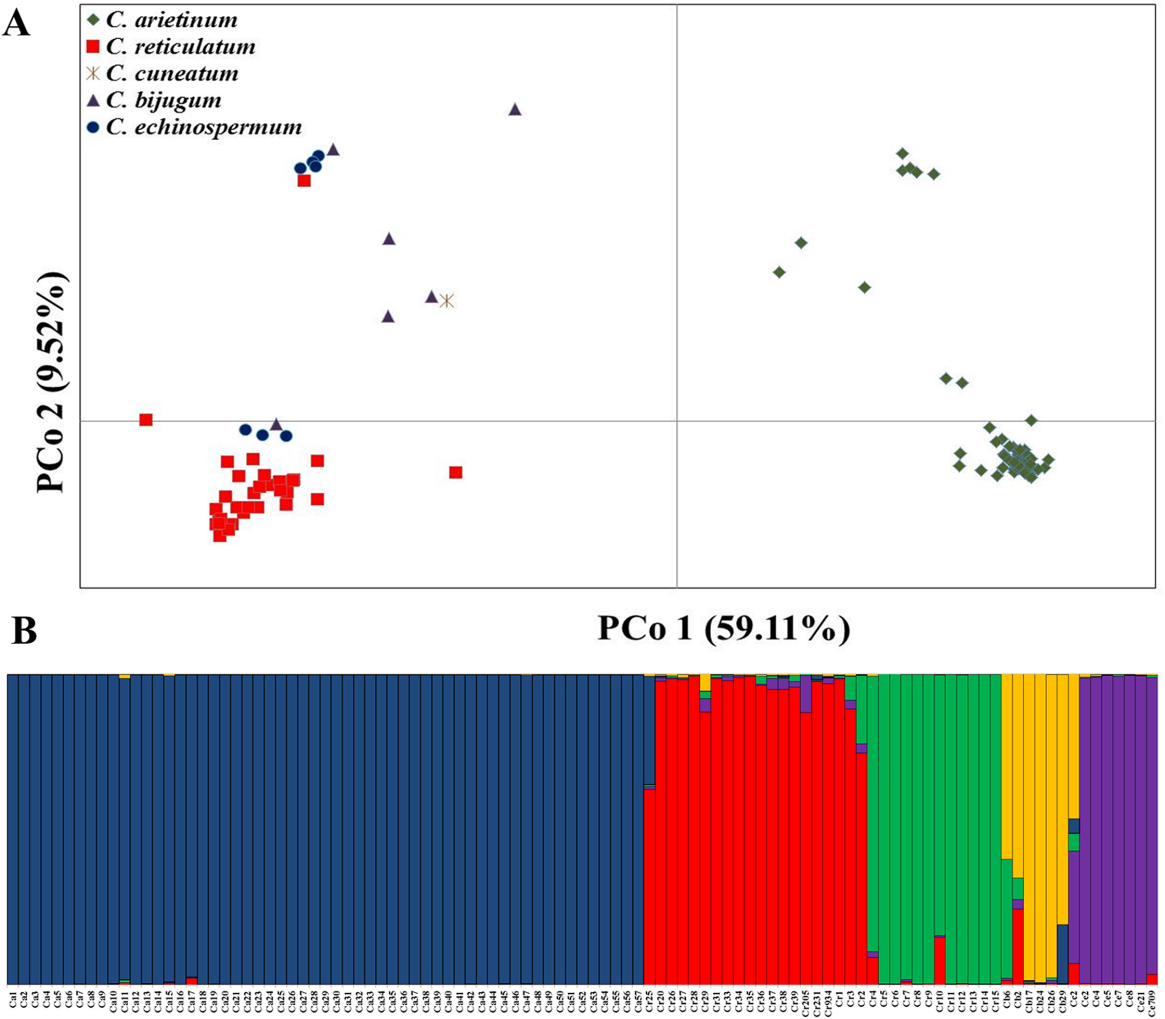
Genetic relationships between *Cicer* sp. (A) A principal coordinate (PCo) plot of pairwise individual genetic distances among four wild *Cicer* species and domesticated genotypes. (B) Estimated population structure based on allele frequency variation of SNPs markers. Each genotype is represented by a vertical bar, which is partitioned into *K-*colored components representing the ancestry fractions in *K* = 5 clusters. Individual genotypes are ordered by species (labeled below).

Genetic relationships among the domesticate cultivars (*C*. *arietinum*) and accessions belonging to four wild species (*C*. *reticulatum*, *C*. *echinospermum*, *C*. *bujugum* and *C*. *cuneatum*) were investigated by principle coordinate analysis (PCoA) constructed from the Dice similarity coefficient matrix [52]. Two principal coordinates, PCo1 and PCo2, accounted jointly for 68.63% of the allelic variation among the studied germplasm (Fig 2A). PCo1 accounted for most of the variation (59.11%) and shows clear separation between the domesticate cultivars and the accessions belonging to the four wild taxa. PCo2 explained only 9.52% of the allelic variation and did not create any clear separation between the taxonomic groups (Fig 2A).

The STRUCTURE analysis performed with domesticated chickpea and the accessions belonging to the four wild species is presented in Fig 2B. The probabilities of the *K* number of clusters showed the best solution for *K* = 5 which was considerably better than *K* = 4, while *K*≥6 gave only a small probability improvement. The lowest level of admixture was observed among the domesticated cultivars (blue, Fig 2B). A very low level of admixture was observed in *C*. *echinospermum* (for accession Ce709), and varying degrees of admixture can be seen in both *C*. *reticulatum* and *C*. *bijugum* (Fig 2B).

**Fig 3:**
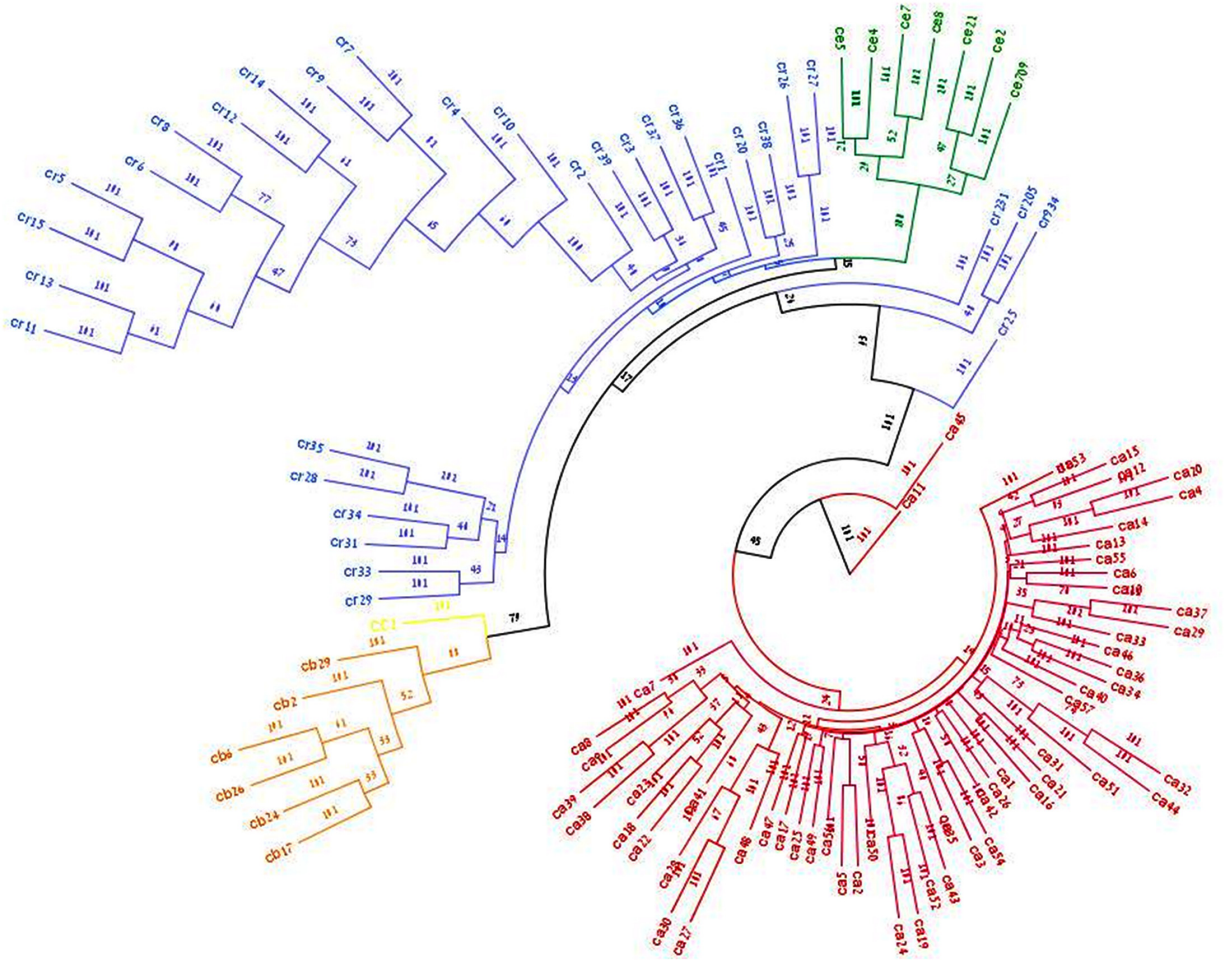
Dendrogrm of *Cicer* sp. UPGMA dendrogram showing the cluster pattern of four wild *Cicer* species and domesticated chickpea based on genetic similarity values obtained from SNPs markers. Genotypes identities can be found in S1 Table. *C*. *arietinum* (red), *C*. *reticulatum* (blue), *C*. *bijugum* (orange), *C*. *echinospermum* (green) and *C*. *cuneatum* (yellow).

A consensus UPGMA unrooted tree based on shared alleles genetic distances between all pairwise combinations of genotypes employed in this study is depicted in Fig 3. Four prominent clusters are apparent from this dendrogram tree. Generally, the detected clusters conform to the known taxonomy of the studied material. All but two domesticated chickpea cultivars fell into one cluster (red, Fig 3), and likewise all *C*. *echinospermum* accessions (green, Fig 3). Similarly, all *C*. *bijugum* accessions formed one cluster (orange, Fig 3). Except from Cr25 that occupies an intermediate position along the main branch separating the cultigen from the wild clusters, all accessions of *C*. *reticulatum* fell into several subgroups (blue, Fig 3). One small group consists of three accessions (Cr205, 231 and 934) holds an intermediate position between the domesticated cultivars and the wild accessions. Another separation was caused by the deviation of the eastern most population from the pattern of the central and western populations (Cr4-15). Another sub-grouping occurred among the central populations of *C*. *reticulatum* sampled near Midyat. A single accession of *C*. *cuneatum* (Cc native to the east African highlands), taken as an out-group, captured an adjacent position near the *C*. *bijugum* cluster.

### Relationship between domesticated chickpea and its wild progenitor

**Fig 4:**
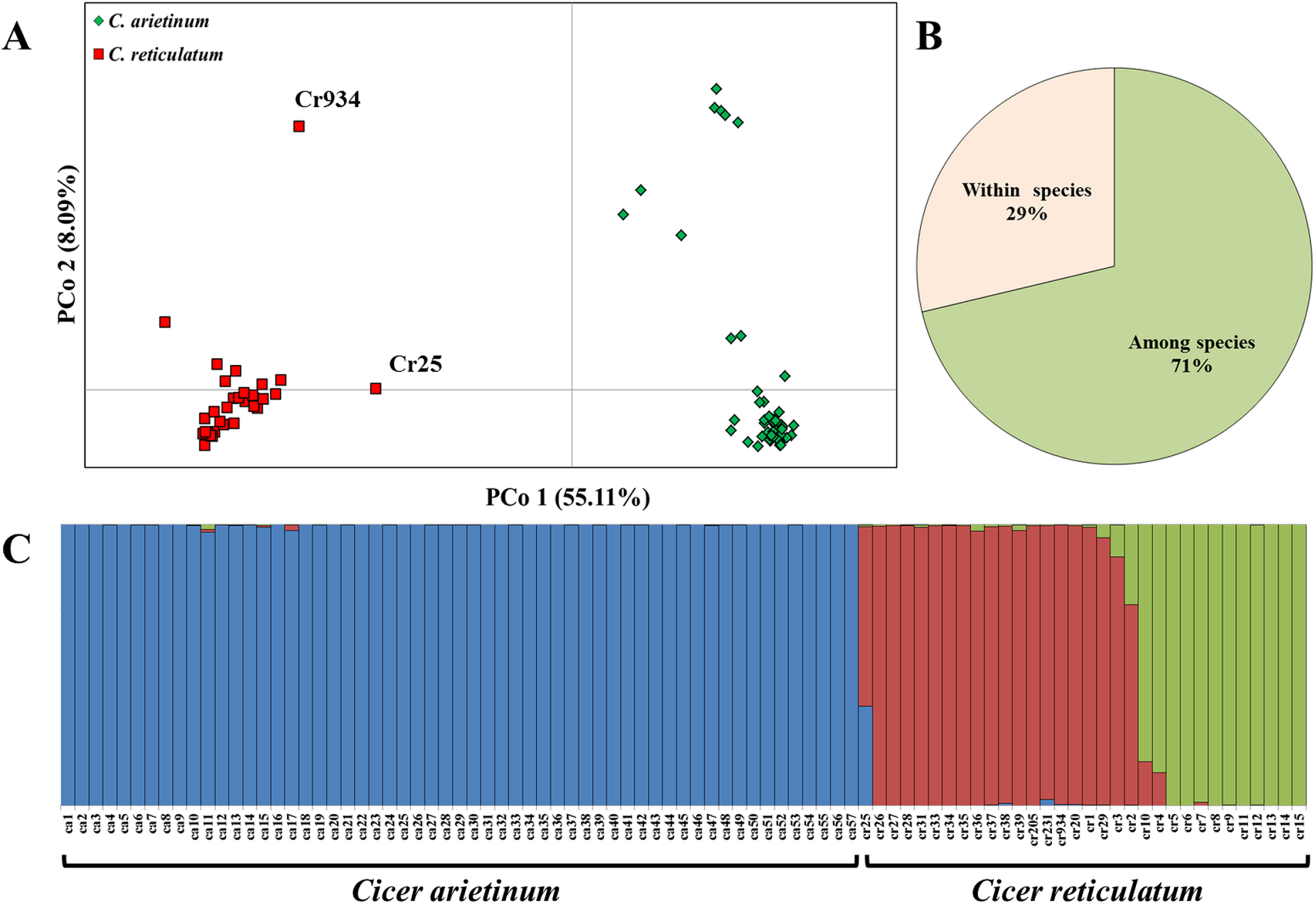
Genetic relationships between wild and domesticated *Cicer* sp. (**A**) A principal coordinate (PCo) plot of pairwise individual genetic distances among wild *Cicer reticulatum* and domesticated *C*. *arietinum* genotypes. (**B**) Estimated population structure based on allele frequency variation of SNPs markers. Each genotype is represented by a vertical bar, which is partitioned into *K-*colored components representing the ancestry fractions in *K* = 3 clusters. Individual genotypes are ordered by species (labeled below).

In order to learn more on the relationship between the domesticated chickpea genepool and its immediate wild progenitors, we have analysed a sub set of the data that belongs to *C*. *arietinum* and *C*. *reticulatum* only. PCoA based on the SNP variation showed two major components explaining jointly 63.2% of the allelic diversity between genotypes (Fig 4A). PCo1 that accounted for 55.11% of the allelic variance and explained most of the separation between the wild and the domesticated germplasm. PCo2 accounted only for 8.09% of the allelic diversity with only one *C*. *reticulatum* accession (Cr934, sampled along the Golbasi-Adiyaman road) separated from the rest of the wild accession along this axis (Fig 4A). However, a number of domesticated cultivars (including Ca12, 17, 24, 26 and 29, with no clear geographic pattern) are separated from the main domesticated cluster along PCo2. The AMOVA shows that 71% of the allelic variation was documented between the domesticated and the wild genepools. The remaining 29% of the allelic variation was observed within the two species (Fig 4B).

**Fig 5:**
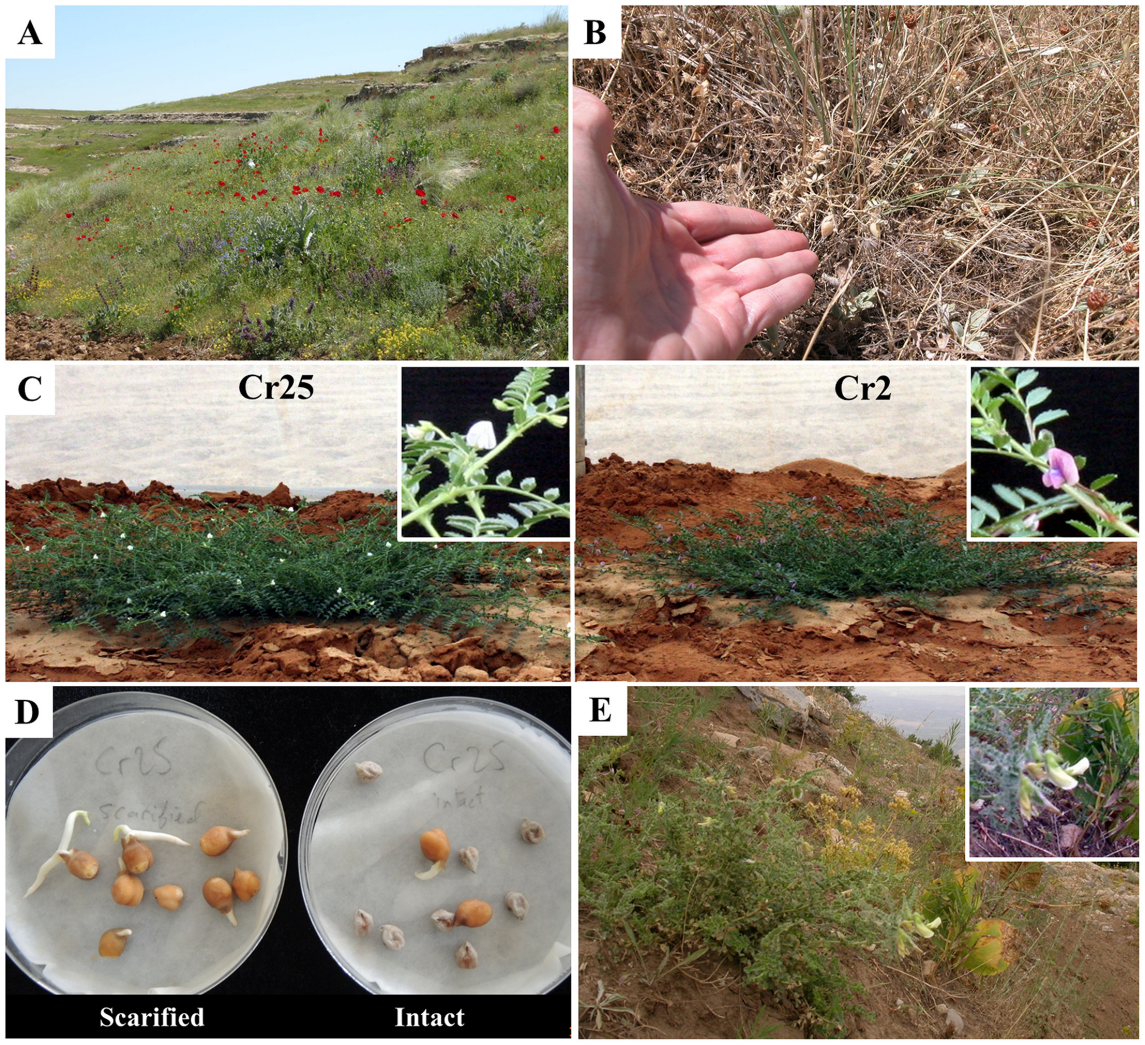
Wild *Cicer reticulatum* accession Cr25. (**A**) The habitat near Midyat, Turkey where Cr25 was sampled, photographed in May 2009. (**B**) A mature wild *C*. *reticulatum* plant in the source population of Cr25 on the occasion of seed sampling, June 2007. (**C**) Growth habit and flower colour of Cr25 as compared with a typical *C*. *reticulatum* accession (Cr2). (**D**) The seed germination pattern of Cr25. Right, intact 5 months old seeds. Left, control (scarified) 5 month old seeds. (**E**) A perennial *Cicer* sp. with white flowers. Photograph taken on Mt. Kizilnora, Uzbekistan, July 2005.

Using this sub-set of the data, the probabilities of the *K* number of clusters showed the best solution for *K* = 3 which was considerably better than *K* = 2 while *K*≥4 gave only a small probability improvement. Low level of admixture was found between domesticated chickpea (blue in Fig 4C) and its wild progenitor *C*. *reticulatum* (red and green in Fig 4C). According to the STRUCTURE plot, a single wild accession sampled near Midyat (Cr25, Figs 1, 2B, 5A and 5B) shows evidence suggesting admixture between wild and domesticated chickpea. Accordingly, this accession (Cr25) holds a median position along the axis connecting the domesticated chickpea cluster and the *C*. *reticulatum* cluster in the phylogenetic tree (Fig 3). The STRUCTURE grouping also shows a separation between the eastern most *C*. *reticulatum* population (12 accessions) sampled between Sirnak and Hakkari, and the remaining populations mostly sampled near Savur and along the Midya–Batman road (red green separation in Fig 4C).

## Discussion

### Genetic relatedness

The dendrogram tree (Fig 3) provides an interesting perspective concerning the genetic affinities between the studied species. The two wild species *C*. *reticulatum* and *C*. *echinospermum* belong to the same crossability group together with the domesticated chickpea [53]. However hybridization experiments and the pattern of meiotic chromosome pairing in interspecific hybrids have shown that *C*. *echinospermum* is more distantly related to the domesticated chickpea as compared with the wild progenitor *C*. *reticulatum* [24], thereby placing *C*. *echinospermum* in the secondary genepool of the cultigen. This however, is not reflected in the position of *C*. *echinospermum* and *C*. *reticulatum* relative to the domesticated accessions in the UPGMA tree (Fig 3). Yet another interesting feature of the UPGMA tree is the relative position of the single *C*. *cuneatum* accession (that was recruited for this analysis to serve as an out-group) adjacent to the *C*. *bijugum* cluster. This may suggest that both species (*C*. *bijugum* and *C*. *cuneatum* are as closely (or distantly) related to the remaining two wild taxa and likewise to the cultigen.

These observations raise questions concerning the role commonly attributed to analyses based on calculations of genetic distances derived from frequencies of shared allele or other methods based on DNA sequence similarity for determining the genetic affinity of crop plants and their wild relatives. For example, contrary to our results, based on the PCoA analysis of Roorkiwal et al. [54] no distinction is seen between the position of the *C*. *echinospermum* accession and the remaining *C*. *reticulatum* accessions and likewise in their STRUCTURE diagram (Fig 4, therein). In addition, and unlike our findings, the phylogenetic tree of Roorkiwal et al. [54] shows a single *C*. *bijugum* accession (ICC17187) well within the primary genepool branch among other *C*. *reticulatum* accessions (Fig 4, therein). So based on the work of Roorkiwal et al.’s [54] one might get the impression that certain *C*. *bijugum* germplasm may have contributed a detectable amount allelic variation to the primary genepool of domesticated chickpea. However, based on the documented crossability relations among the annual *Cicer* sp. this is highly unlikely [53]. A recent attempt to identify the wild ancestry of domesticated lentil faced similar problems. The results of the DNA sequence analyses have inspired Alo et al. [55] to propose a revision of *Lens* sp. taxonomy including a suggestion to group together several cross-incompatible distinct biological species as sub-specific forms into a single taxonomic unit.

Phylogenetic analyses based on DNA markers and sequence comparisons or biochemical markers constitute a powerful biological tool and may provide deep evolutionary insights. Often such analyses may assist in resolving germplasm misclassifications. For example, while exploring genetic diversity among *Cicer* sp. Nguyen et al. [56] have noted that a single alleged *C*. *reticulatum* accession (ATC42326) was placed away from the remaining *C*. *reticulatum* accessions and next to *C*. *echinospermum* accessions (p. 175–176, therein). ATC42326 is identical with an accession maintained by the USDA as PI 593709 [57]. While working with this germplasm line (Fig 3 herein, denoted Cr709) more than 10 years ago, we have noticed that its morphology and especially its seed coat pattern do not conform to that of *C*. *reticulatum* but rather fits the description of *C*. *echinospermum*. Indeed, in both the present study and the work of Nguyen et al.’s [56], the DNA based dendrogram was in full match with the morphology. Still, in other instances (mentioned above) the obtained DNA phylogenetic patterns do not make sense regarding the known biology of the studied groups. Therefore, analyses based on relative genetic distances should always be considered alongside and in the context additional information including (but not limited to) morphology, crossability relations, chromosome pairing patterns in F_1_ hybrids, karyotypic variation and ecological affinity (e.g., [24, 34, 58, 59]).

### Genetic relationship between domesticated chickpea and its wild progenitor

Both the PCoA and STUCTURE analyses (Fig 4A and 4C) provided a clear separation between the domesticated chickpea cultivars and the accessions sampled across the distribution range of the wild progenitor *C*. *reticulatum*. The overall pattern does suggest a monophyletic origin of the cultigen (Fig 3). A small group of *C*. *reticulatum* accession [two sampled near Savur (Cr205, 231) and one sampled near Golbasi (Cr934)] seem as likely members of the wild stock of the domesticated cultivars. However, the placement of these three germplasm lines in the dendrogram tree occurred at relatively low likelihood as reflected with the low bootstrap values. It should be borne in mind that the available germplasm most probably captures only part of the entire ecogeographic range of this species. We are aware of efforts to increase the number of wild *Cicer* accessions by various research groups. In conjunction with the rapid accumulation of genomic sequence and polymorphism data [60–63], these efforts are likely to create an impact in the near future. Therefore, no firm conclusions concerning the wild stock of chickpea can be made at this stage.

Based on agronomic considerations, and the limited genetic data available at the time, Abbo et al. [64] have pointed out the likely reasons for the relatively narrow agro-ecologic adaptation and low genetic diversity in the domesticated chickpea genepool. These include the limited ecological amplitude of the wild progenitor as well as two ancient evolutionary ‘bottlenecks’ one presumably associated with the domestication ‘Founder Effect’ [65], and another with the selection under domestication for vernalization insensitivity [64, 66, 67]. Indeed, the known sites of *C*. *reticulatum* populations are situated along a rather narrow latitudinal range (Fig 1), a likely reflection of inherent relatively narrow adaptation. Our PCoA and STRUCTURE analyses (Fig 4A and 4C) strongly support the hypotheses of Abbo et al. [64] concerning the limited diversity among both the domesticated and wild genepools of chickpea.

While some authors consider the natural distribution area (or parts of it) of the wild progenitors as the likely arena of Near Eastern plant domestication (e.g., [14, 40, 41, 68]), others assert that domestication could not have been achieved within the range of the wild relatives, and therefore must have occurred at its periphery or outside it (e.g., [33, 69]). Assumptions concerning potential geneflow from the wild relatives into the nascent crops were advanced among other arguments in support of domestication at the periphery (or outside) of the natural range of the wild progenitor (e.g., [69]). Interestingly enough, no data was ever presented to support or refute such suggestions concerning the Near Eastern crops. In the present work we show, for the first time, evidence suggestive of geneflow from domesticated cultivars into wild populations in chickpea. Accession Cr25, collected near Midyat, Turkey (Fig 5A and 5B), shows considerable level of admixture (Fig 4C). This accession has white flowers (Fig 5C), lightly reticulated seed coat (Fig 5D), and its growth habit is not as prostrate as other typical *C*. *reticulatum* accessions (Fig 5C). These traits may have been contributed through introgression from domesticated genotype(s). This accession has dehiscent pods and *wild type* germination pattern (Fig 5C), two highly important adaptive traits among Near Eastern grain legumes [65, 70, 71] that may explain its survival in the wild population.

White flower is certainly among the naturally occurring variation of wild *Cicer* spp. as evident from its occurrence in perennial *Cicer* sp. population documented on Mt. Kizilnora, Uzbekistan (Fig 5E). However, white flower occurrence in a *C*. *reticulatum* population accompanied by lower seed coat reticulation and a considerable admixture with domesticated alleles suggest that in this case it is more likely to have been a result of wild-domesticated introgression. Of note is a domesticated accession, Ca11 that was sampled within the distribution range of *C*. *reticulatum* (near Ömerli) that shows no sign of wild alleles infusion and likewise other tested Turkish accessions (listed in S1 Table).

This pattern may suggest that ancient farmers were (and likewise present day farmers are) fully capable of maintaining the desired phenotypes of their crops despite inevitable introgressions when crop plants and their wild progenitors grow sympatrically. This was elegantly demonstrated in terms of asymmetric geneflow between wild and domesticated common bean [72], and for wild-domesticated-feral *Phaseolus* complexes, both in Mexico [73]. Moreover, if maintenance of desired domesticated phenotypes is possible for a wind pollinated crop like maize (e.g., [74–76]), this must have been much simpler for the Neolithic Near Eastern domesticators that mostly dealt with self-pollinating annual species.

### Implication for germplasm conservation

The traditional farming systems in southeastern Turkey are situated at the ‘Core Area’ of the Near Eastern Neolithic agriculture. In this region domesticated lentil, chickpea, bitter vetch, and emmer wheat are grown in sympatry with their immediate wild progenitors (e.g., [41]). As such, both the authentic landraces of the area as well as the wild populations are exposed to introgression events. Moreover, some of the habitats of wild relatives of the abovementioned crop plants are in fact within arable land (e.g., [76]). This applies (but may not be limited to) *C*. *echinospermum* and *C*. *bijugum* that have strong affinity to deep basaltic vertisols that at present are mostly ploughed in that region, as well as to wild einkorn (*Triticum boeoticum*) and wild rye (*Secale* sp.). Therefore, given the fact that in modern times, the size of the natural populations is constantly being reduced relative to the permanent increase of arable land, a special care should be taken by policy makers to ensure the existence of large enough protected areas to enable sustainable survival of wild relatives that have patchy and thin populations (e.g., *Pisum* sp. and *Cicer* sp.). The potential for domesticated into wild geneflow should also be carefully considered in discussions concerning introduction of GM crop varieties into such vulnerable farming systems.

## Conclusions

The traditional farming systems of outheastern Turkey are characterized by occurrence of sympatric wild progenitor—domesticated forms of chickpea (and likewise cereals and other grain legumes). Therefore, both the authentic crop landraces and the wild populations native to the area are a unique genetic resource. Our results grant support to the notion of domestication within the natural distribution range of the wild progenitor, suggesting that the Neolithic domesticators were fully capable of selecting the desired phenotypes even when facing rare wild-domesticated introgression events.

## Supporting Information

S1 Table List of genotypes used for the current study and SNPs score. (XLSX)
